# Comprehensive adipocytic and neurogenic tissue microarray analysis of NY-ESO-1 expression - a promising immunotherapy target in malignant peripheral nerve sheath tumor and liposarcoma

**DOI:** 10.18632/oncotarget.12096

**Published:** 2016-09-17

**Authors:** Elizabeth Shurell, Maria E. Vergara-Lluri, Yunfeng Li, Joseph G. Crompton, Arun Singh, Nicholas Bernthal, Hong Wu, Fritz C. Eilber, Sarah M. Dry

**Affiliations:** ^1^ Division of Surgical Oncology, University of California, Los Angeles, CA 90095, USA; ^2^ Department of Pathology and Laboratory Medicine, University of California, Los Angeles, CA 90095, USA; ^3^ Department of Molecular and Medical Pharmacology, University of California, Los Angeles, CA 90095, USA; ^4^ Department of Hematology/Oncology, University of California, Los Angeles, CA 90095, USA; ^5^ Department of Orthopaedic Surgery, University of California, Los Angeles, CA 90095, USA

**Keywords:** NY-ESO-1, sarcoma, MPNST, liposarcoma, immunotherapy

## Abstract

**Background:**

Immunotherapy targeting cancer-testis antigen NY-ESO-1 shows promise for tumors with poor response to chemoradiation. Malignant peripheral nerve sheath tumors (MPNSTs) and liposarcomas (LPS) are chemoresistant and have few effective treatment options.

**Materials Methods:**

Using a comprehensive tissue microarray (TMA) of both benign and malignant tumors in primary, recurrent, and metastatic samples, we examined NY-ESO-1 expression in peripheral nerve sheath tumor (PNST) and adipocytic tumors. The PNST TMA included 42 MPNSTs (spontaneous *n* = 26, NF1-associated *n* = 16), 35 neurofibromas (spontaneous *n* = 22, NF-1 associated *n* = 13), 11 schwannomas, and 18 normal nerves. The LPS TMA included 48 well-differentiated/dedifferentiated (WD/DD) LPS, 13 myxoid/round cell LPS, 3 pleomorphic LPS, 8 lipomas, 1 myelolipoma, and 3 normal adipocytic tissue samples. Stained in triplicate, NY-ESO-1 intensity and density were scored.

**Results:**

NY-ESO-1 expression was exclusive to malignant tumors. 100% of myxoid/round cell LPS demonstrated NY-ESO-1 expression, while only 6% of WD/DD LPS showed protein expression, one of which was WD LPS. Of MPNST, 4/26 (15%) spontaneous and 2/16 (12%) NF1-associated MPNSTs demonstrated NY-ESO-1 expression. Strong NY-ESO-1 expression was observed in myxoid/round cell and dedifferentiated LPS, and MPNST in primary, neoadjuvant, and metastatic settings.

**Conclusions:**

We found higher prevalence of NY-ESO-1 expression in MPNSTs than previously reported, highlighting a subset of MPNST patients who may benefit from immunotherapy. This study expands our understanding of NY-ESO-1 in WD/DD LPS and is the first demonstration of staining in a WD LPS and metastatic/recurrent myxoid/round cell LPS. These results suggest immunotherapy targeting NY-ESO-1 may benefit patients with aggressive tumors resistant to conventional therapy.

## INTRODUCTION

Targeted immunotherapy presents an opportunity to overcome chemotherapeutic resistance in several human cancers. Cancer-testis antigen NY-ESO-1 (New York esophageal squamous cell carcinoma 1), encoded by the *CTAG 1B* gene, has quickly evolved into a vaccine and immunotherapy candidate since its identification in the late 1990's. Initially detected in the serum of patients with esophageal squamous carcinoma [1], NY-ESO-1 is present in fetal and adult germ cells and is not detectable in somatic tissues (excluding insignificant expression in brain, thymic, uterine and placental tissue), making it an attractive cancer immunotherapy target [2]. An appealing feature of NY-ESO-1 is its ability to elicit spontaneous antibody and T-cell responses in certain cancer subtypes. Currently, NY-ESO-1 is the focus of adoptive cellular therapy and vaccine trials for melanoma, sarcoma, multiple myeloma, non-small cell lung carcinoma, and urothelial, esophageal, ovarian, and prostatic neoplasms [3–5].

Amongst sarcomas, synovial sarcomas and myxoid/round cell liposarcomas frequently express NY-ESO-1. Recently, Robbins *et al.* published an adoptive therapy trial using retrovirally transfected, NY-ESO-1–specific T-cell receptor, and documented clinical responses in 66% of patients with synovial sarcoma [6]. However, to date, limited numbers of peripheral nerve sheath tumors (PNSTs) and non-myxoid/round cell liposarcomas (LPS) have been tested. Malignant peripheral nerve sheath tumors (MPNST), in particular, portend a poor patient prognosis and show limited response to traditional chemoradiation [7–11]. Therefore, investigation into synergistic therapies is warranted.

The purpose of this study was to characterize the prevalence and intensity of NY-ESO-1 in PNSTs and adipocytic neoplasms. In order to include large numbers of different tumor subtypes, as well as primary, locally recurrent, and metastatic tumors, we utilized previously constructed tissue microarrays (TMAs) of PNSTs and adipocytic tumors from our institution. To our knowledge, this is the first report that specifically evaluates NY-ESO-1 expression in primary, recurrent, and metastatic MPNSTs and LPS. This is also the first demonstration of differential staining in spontaneous and NF1-associated PNSTs, and in benign neurofibromas.

## RESULTS

Strong NY-ESO-1 expression was observed in samples of myxoid/round cell LPS, dedifferentiated LPS, and MPNST in primary, neoadjuvant, and metastatic settings (See Table 1, Table 2).

**Table 1:** NY-ESO immunohistochemical results

	*N*	NY-ESO-1 Positive (% of total)
**Adipocytic Tumors**		
Myxoid/round cell LPS	13	13 (100%)
Well-differentiated/Dedifferentiated LPS	48	3 (6%)
Pleomorphic LPS	3	0 (0%)
Lipoma	8	0 (0%)
Myelolipoma	1	0 (0%)
Total	73	16 (22%)
**Peripheral Nerve Sheath Tumors**		
Spontaneous MPNSTs	26	4 (15%)
NF1-associated MPNSTs	16	2 (12%)
Spontaneous NFs	22	0 (0%)
NF1-associated NFs	13	0 (0%)
Cellular Schwannoma	11	0 (0%)
Total	88	6 (7%)
**Normal tissues**		
Nerve	18	0 (0%)
Fat	3	0 (0%)

**Table 2:** Characteristics of patients with positive NY-ESO-1 expression and staining pattern

Patient Number	Tumor Type	Tumor Location	Tumor at Presentation	Neoadjuvant/ Adjuvant Therapy	NY-ESO-1 density	NY-ESO-1 intensity
4	Myxoid LPS	Thigh	Recurrent	no	3	3+
8	Myxoid LPS	Gluteal	Primary	no	3	2+
10a	Myxoid LPS	Thigh	Primary	no	3	3+
10b	Myxoid LPS	Thigh	Recurrent	no	1	2+
14	Myxoid LPS	Thigh	Primary	no	3	3+
17	Myxoid LPS with 5–10% round cell component	Thigh	Primary	no	3	3+
26	Myxoid LPS	Groin	Metastatic	yes	3	3+
29	Myxoid LPS with predominant round cell component	Thigh	Primary	yes	3	3+
31	Myxoid LPS	Gluteal	Primary	yes	3	3+
33a	Myxoid LPS	Thigh	Primary	no	2	3+
33b	Myxoid LPS	Abdomen	Metastatic	no	3	3+
46	Myxoid LPS	Thigh	Primary	no	3	3+
54a	Myxoid LPS	Gluteal	Primary	no	3	3+
54b	Myxoid LPS	Shoulder	Metastatic	yes	3	3+
54c	Myxoid LPS	Paraspinal	Metastatic	yes	3	3+
71a	Myxoid LPS	Calf	Primary	no	3	2+
71b	Myxoid LPS	Epidural	Metastatic	yes	3	3+
71c	Myxoid LPS	Abdomen	Metastatic	yes	1	3+
71d	Myxoid LPS	Bone, T11-T12	Metastatic	yes	3	3+
71e	Myxoid LPS	Sacrum	Metastatic	yes	2	3+
73	Myxoid LPS	Thigh	Primary	yes	3	3+
6	DD LPS arising from WD LPS (only WD seen)	Retroperitoneum	Recurrent	no	3	3+
32	DD LPS arising from WD LPS (only DD seen)	Leg	Primary	no	2	3+
64	DD LPS	Groin	Recurrent	yes	3	1+
105	sMPNST, high grade	Leg	Primary	no	2	2–3+
109	sMPNST, high grade	Thigh	Primary	no	1	1+
121	sMPNST, high grade	Thigh	Primary	yes	3	3+
152	sMPNST, high grade	Forearm	Primary	yes	3	3+
129	NF1- associated MPNST v. Post-XRT sarcoma	Neck	Recurrent	yes	2	1+
110	NF1-MPNST, high grade	Thigh	Primary	yes	3	3+

### Lipomatous tumors

**Figure 1:**
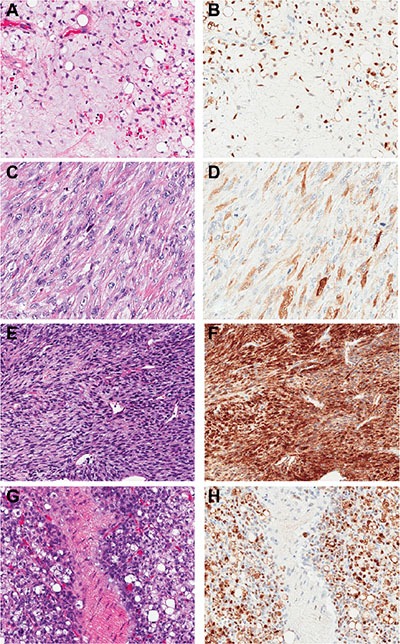
NY-ESO-1 immunohistochemical staining pattern and intensity in liposarcomas and MPNST Primary myxoid/round cell liposarcoma, H&E (**A**) and diffuse (> 75% of cells staining) 3+ IHC positivity (**B**). Dedifferentiated liposarcoma, H&E (**C**) and 3+ IHC positivity in > 25% of cells (**D**). MPNST, H&E (**E**) and diffuse 3+ IHC positivity (**F**). Metastatic mxyoid/round cell liposarcoma, H&E (**G**) and diffuse 3+ IHC positivity (**H**). (200× magnification).

All thirteen patients (100%) with myxoid/round cell liposarcoma exhibited strikingly uniform, moderate to intense (2–3+) NY-ESO-1 expression with a clean background devoid of artefactual staining (Table 2). Eleven of these thirteen cases were primary tumors. All primary tumors (11/11) demonstrated moderate to intense (2–3+) positivity which was diffuse (> 75% of tumor cases). Three of the primary myxoid/round cell liposarcoma cases had received neoadjuvant chemotherapy; all post-treatment tumors showed diffuse, strong staining for NY-ESO-1. A representative example of a primary myxoid/round cell liposarcoma and its staining pattern are shown in Figure 1A and 1B.

Four patients with myxoid/round cell liposarcoma had multiple tumors, i.e. both primary and recurrent and/or metastatic tumors from the same patient represented on the TMA [patients #10, 33, 54, 71]. These 4 patients had a total of eight recurrent or metastatic tumors. All eight tumors continued to display moderate to strong (2–3+) staining intensity, similar to their primary tumors. One patient (#33) exhibited an increase in the percentage of NY-ESO-1 positive tumor cells in the recurrent/metastatic tumors from focal (< 25% of tumor cells) to diffuse (> 75% of tumor cells) (Table 3) (Figure 1H). Staining in recurrent and metastatic tumors generally showed similar or an increased intensity or density of staining as compared to the primary tumor (Table 3).

**Table 3:** Comparison of changes in expression patterns of primary, locally recurrent, and metastatic tumors

Patient Number	Tumor Type	Tumor	Location of Tumor Recurrence/Metastasis	Staining Density Changes	Staining Intensity Changes
10	Myxoid LPS	Primary **→** Recurrent	Thigh	Decreased (3 → 1)	Decreased (3+ → 2+)
54	Myxoid LPS	Primary → Metastatic	Shoulder	Unchanged (3 → 3)	Unchanged (3+ → 3+)
	Myxoid LPS	Primary → Metastatic	Paraspinal	Unchanged (3 → 3)	Unchanged (3+ → 3+)
71	Myxoid LPS	Primary → Metastatic	Epidural	Unchanged (3 → 3)	Increased (2+ → 3+)
	Myxoid LPS	Primary → Metastatic	Abdomen	Decreased (3 → 1)	Increased (2+ → 3+)
	Myxoid LPS	Primary → Metastatic	Bone, T11-T12	Unchanged (3 → 3)	Increased (2+ → 3+)
	Myxoid LPS	Primary → Metastatic	Sacrum	Decreased (3 → 2)	Increased (2+ → 3+)
33	Myxoid LPS	Primary → Metastatic	Abdomen	Increased (2 → 3)	Unchanged (3+ → 3+)

Among the well differentiated/dedifferentiated liposarcomas, 3 of 48 (6%) of tumors exhibited NY-ESO-1 expression. One showed positive staining in the well-differentiated component and two in the dedifferentiated component. Of these three positive tumors, 1 was a primary presentation of a well differentiated/dedifferentiated liposarcoma, and 2 were recurrent tumors. Two of these, a recurrent retroperitoneal tumor (patient #6 – well differentiated component only) and a primary leg tumor (patient #32 - dedifferentiated component only), exhibited 3+ intense staining in > 75% and > 25% of cells, respectively (Figure 1C and 1D). The third case was a recurrent groin dedifferentiated liposarcoma that showed weak (1+) yet diffuse (> 75% of tumor cells) staining (patient #64). (See Table 2) None of the pleomorphic liposarcomas revealed any NY-ESO-1 expression, nor did any of the benign lipomatous tumors or normal adipocytic tissue.

### Peripheral nerve sheath tumors

Fourteen percent of MPNSTs expressed NY-ESO-1, mainly in spontaneous MPNST cases [4 of 26 (15%) spontaneous MPNSTs and 2 of 16 (12%) NF1-associated MPNSTs]. All four of the spontaneous MPNSTs and one of the NF1-associated tumors were primary neoplasms and the other NF-1 patient had a recurrent tumor. The expression patterns of the MPNSTs were highly variable. Three of the spontaneous MPNSTs showed moderate to strong staining intensity (2–3+), with diffuse staining in most samples. (Table 2, Figure 1E and 1F) Only one spontaneous MPNST displayed weak (1+) and focal (< 25% of tumor cells) expression of NY-ESO-1. The two NF1 patients showed relatively diffuse tumoral staining (density score 2–3), but the intensity varied from weak (1+) in the recurrent tumor (patient #129) to strong (3+) in the primary thigh NF1-MPNST. No spontaneous or NF-1 associated neurofibromas (0/35), schwannomas (0/11), or normal nerves expressed NY-ESO-1.

## DISCUSSION

To our knowledge, this report significantly expands the PNSTs and well differentiated/dedifferentiated liposarcomas tested for NY-ESO-1, including a total of 42 MPNSTs (both NF1-associated and sporadic tumors) and 64 liposarcomas. It also is the first study to investigate NY-ESO-1 immunostaining patterns in primary, recurrent, and metastatic myxoid/round cell liposarcomas.

Recently, Lai and colleagues demonstrated NY-ESO-1 staining in 76% of synovial sarcomas, as well as positivity in limited numbers of other sarcomas including 2.9% (1/34) of MPNSTs and a single case (1/1) of dedifferentiated liposarcoma [12]. In their cohort, the striking prevalence of a strong and diffuse pattern of NY-ESO-1 staining (defined as 2–3+, > 50–70% of tumor cells) appeared to be confined to synovial sarcomas, prompting them to hypothesize that it may be a useful marker in the differential diagnosis with other spindle cell neoplasms [12]. However, in the current study, we evaluated a larger number of MPNSTs, both spontaneous and NF1-associated, and have shown that a higher percentage (14%) of MPNSTs displayed positivity, including 67% with intense (2−3+) staining than had been previously described. This result suggests caution in relying on NY-ESO-1 as a confirmatory stain to distinguish synovial sarcoma from other spindle cell neoplasm subtypes as Lai *et al*. suggested.

Hemminger *et al* and Pollack *et al.* found NY-ESO-1 to be very highly expressed in myxoid/round cell liposarcomas, with 89% and 100% expression, respectively [13, 14]. Indeed, this observation holds true in the current study, wherein we confirm 100% of myxoid/round cell liposarcoma cases showed moderate to strong (2–3+) staining for NY-ESO-1. Our study demonstrated, for the first time, that all recurrent and metastatic myxoid/round cell liposarcomas in our series retained their moderate to intense NY-ESO-1 expression pattern. Furthermore, three of these tumors continued to show strong positive staining following treatment with neoadjuvant chemotherapy.

Our results also suggest possible new treatments for MPNST, well-differentiated liposarcoma and dedifferentiated liposarcoma, all of which respond poorly to existing chemoradiation therapies (6–10). Once patients with dedifferentiated liposarcoma develop non-visceral and visceral metastases, their median progression-free survival is 6.1 months and 3.3 months, respectively, and median overall survival 17.2 and 11.1 months, respectively [11] despite therapy. Recently NY-ESO-1 adoptive cellular therapy was granted orphan drug designation for synovial sarcomas and further confirmatory studies are ongoing [6, 15]. Future studies will be needed to determine if NY-ESO-1 based immunotherapy shows clinical response in MPNST and well differentiated/dedifferentiated liposarcoma patients, and to identify the ideal histologies in which NY-ESO-1 immunotherapy may be particularly effective.

Murine models of MPNST and liposarcoma can provide a platform for the testing of novel therapies and further investigation of the potential of adoptive cell therapy in the treatment of sarcoma [16–21]. Guo *et al.* (2006) induced cancer testis antigen in three murine sarcoma cell lines *in vitro*, and in the MCA102 derived murine xenograft model *in vivo* using 5-Aza-2′-Deoxycytidine [22], suggesting that induction of cancer testis antigen may be possible in sarcoma patients whose tumors may not have robust expression of NY-ESO-1. Immunogenic tumor-associated antigens, such as NY-ESO-1, that can be induced in sarcoma may be an effective means to break antigen-specific tolerance, which is a major barrier to immune-mediated cancer regression. This study highlights the potential of murine xenograft and transgenic models in the evaluation of adoptive cell therapy in MPNST and liposarcoma.

The enthusiasm around NY-ESO-1 as a potential immunotherapeutic target is not unfounded. Previous testing of immunotherapy regimens generally have shown objective tumor regression in up to 72% of patients with metastatic melanoma who received *in-vitro* cultured melanoma-reactive T cells [23, 24]. Recently, Robbins and colleagues used genetically engineered lymphocytes reactive for NY-ESO-1 in 17 patients with synovial sarcomas and melanomas that were immunohistochemically positive for NY-ESO-1; they demonstrated significant tumor regression in 4 of 6 patients (67%) with synovial sarcoma and 5 of 11 patients (45%) with melanoma. This included objective clinical responses in patients with progressive disease and tumors previously refractory to conventional therapy. Sustained partial response was noted in one of the patients with synovial sarcoma and complete regression was seen in two patients with melanoma [6]. With growing evidence of diffuse and strong expression of NY-ESO-1 in myxoid/round cell liposarcoma, as well as expression in a subset of MPNSTs and well differentiated/dedifferentiated liposarcoma, it certainly provides further support for extending clinical trials to patients with sarcomas that express NY-ESO-1 [14]. Furthermore, adoptive immunotherapy targeting NY-ESO-1 appears to be quite well tolerated, with side effects attributable mainly to the preconditioning regimen [6, 25].

We corroborate the finding that NY-ESO-1 staining is limited to malignant neoplasms and testis [25, 26] and does not appear to be expressed in other normal adult tissues. We also did not find any staining in benign neoplasms, including schwannomas, neurofibromas (spontaneous or NF-1 associated), lipomas, or myelolipomas.

A limitation of this study is the small sample size for the patients (*n* = 4) with multiple recurrent/metastatic myxoid/round cell liposarcoma tumors (a total of 12 separate primary, recurrent, and metastatic tumors). However, the remarkably consistent finding of intense immunopositivity in all the cases of myxoid/round cell liposarcoma is compelling and warrants validation with additional studies.

In summary, this is the first report of strong consistent staining in multiple metastatic and recurrent myxoid/round cell liposarcoma. This study also significantly expands the total number of well-differentiated/dedifferentiated liposarcomas and MPNSTs (both NF-1 associated and sporadic) tested and is the first report of staining in a well differentiated liposarcoma. Our results suggest that NY-ESO-1 targeted immunotherapy may benefit patients with metastatic and recurrent myxoid/round cell liposarcoma. Additionally, a proportion of patients with MPNSTs and well differentiated/dedifferentiated liposarcomas tumor types with historically poor response to chemoradiation may also benefit from NY-ESO-1 targeted immunotherapy. Further clinical trials will help establish the effectiveness of this therapeutic approach in these tumor types.

## MATERIALS AND METHODS

The study was approved by the University of California at Los Angeles Institutional Review Board. Two tissue microarrays (TMA) were constructed at our institution, one with samples of benign and malignant PNSTs and normal nerve and the other with different types of adipocytic tumors and normal adipocytic tissue. Prior to TMA construction, all original cases, as well as any prior or subsequent local recurrences and metastases, were reviewed to confirm the diagnosis by a pathologist with expertise in soft tissue tumors (SD). The comprehensive PNST TMA was constructed from 106 patient samples, including 26 spontaneous MPNSTs, 16 NF1-associated MPNSTs, 22 spontaneous neurofibromas, 13 neurofibromas arising in patients with diagnosed NF-1, 11 spontaneous schwannomas, and 18 samples of normal nerve. The adipocytic TMA was comprised of well-differentiated/dedifferentiated liposarcoma (*n* = 48), myxoid/round cell liposarcoma (*n* = 13), pleomorphic liposarcoma (*n* = 3), lipomas (*n* = 8), myelolipoma (*n* = 1), and normal adipocytic tissue (*n* = 3). Dedifferentiated liposarcomas were diagnosed using the criteria of Evans [27]. Both TMAs contained primary, locally recurrent and metastatic malignant tumors; 9 MPNST and 20 liposarcoma patients had samples drawn from their primary and/or recurrent tumor, and then later developed locally recurrent and/or metastatic disease which were also sampled and used in the TMAs. Patients were treated with neoadjuvant or adjuvant chemotherapy according to NCCN guidelines (Table 2) [28]. Each sample had triplicate core samples to account for tumor heterogeneity.

Each TMA was stained with hematoxylin and eosin stain for morphologic evaluation, as well as NY-ESO-1 immunoperoxidase for immunohistochemical (IHC) scoring. Affinity-purified mouse monoclonal antibody against NY-ESO-1, clone E978 (Sigma- Aldrich, N2038) was used on slides obtained from human FFPE samples, guide dilution of 1:300, and incubated at 4°C overnight. Biotinylated anti-mouse IgG (H+L) antibody (Vector Laboratory, BA-9200) and Horseradish Peroxidase Avidin D (Vector Laboratory, A-2004) were used as Streptavidin-biotin-peroxidase complex (SABC) technique for immunohistochemical staining. Slides were developed in DAB substrate and counterstained with Hematoxylin. Citrate buffer (pH 6.0) for antigen retrieval was used and steamed at 100°C for 30 minutes. NY-ESO-1 staining of tumor cells was scored according to intensity (weak, 1+; moderate, 2+; strong, 3+) and density (1 = 1–25% of tumor cells; 2 = 26–75%; and 3 = > 75%). Examining both tumor intensity and density characterizes tumor samples with low tumor cell density with edematous hyalinized tissue, coagulative necrosis, reactive matrix, and weak NY-ESO-1 staining below the intensity required to label the tumor as positive, as can be seen in post-neoadjuvant treated tumor samples. Cores were doubly scored by two pathologists (M V-L and SD) who were blinded to the diagnosis and tumor status (primary, recurrence, metastatic).
